# Determination of the Efficiency of Hot Nano-Grinding of Mono-Crystalline Fcc Metals Using Molecular Dynamics Method

**DOI:** 10.3390/mi13030415

**Published:** 2022-03-06

**Authors:** Nikolaos E. Karkalos, Angelos P. Markopoulos

**Affiliations:** Laboratory of Manufacturing Technology, School of Mechanical Engineering, National Technical University of Athens, Heroon Polytechniou 9, 15780 Athens, Greece; amark@mail.ntua.gr

**Keywords:** nanogrinding, abrasive processes, molecular dynamics, hot machining

## Abstract

Abrasive processes are essential to the manufacturing field, due to their capability of rendering high-quality surfaces with minimum effect on workpiece integrity. As it is especially difficult to perform sufficient experimental work, numerical studies can be successfully employed to evaluate techniques for the improvement of the efficiency of nanometric abrasive processes. In the present study, for the first time, cases of nanogrinding on workpieces of three different fcc metals, namely, copper, nickel, and aluminum are investigated under different preheating temperatures, in order to determine the efficiency of the hot nano-grinding technique. For the simulations, a molecular dynamics model for peripheral nanogrinding is developed including multiple abrasive grains and realistic grain trajectory and grinding forces, and chip characteristics and subsurface alterations are evaluated. The results indicate that using elevated preheating temperatures is beneficial for nanogrinding, as forces can be considerably reduced and material removal can be facilitated, especially for temperatures over 40% of the material melting temperature (T_m_). However, the detrimental effect on workpiece integrity is also evident at higher preheating temperatures, due to the high temperature on the whole workpiece, posing limitations to the applicability of the hot nano-grinding technique. Based on the findings of this study, preheating temperatures in the range of 0.4–0.55 T_m_ are recommended.

## 1. Introduction

During the last few decades, the production of miniaturized parts has become one of the most promising trends in the field of manufacturing, due to the increased need for microscopic devices in various high-end industrial sectors such as microelectronics, microfluidics, and the biomedical industry, among others. Abrasive processes such as grinding or polishing constitute a promising alternative for more established methods in the micro and nanoscale such as those involving a high energy beam in order to produce high-quality surfaces. Moreover, another important consideration is that production of these microparts should be highly effective and sufficiently profitable. Thus, manufacturing processes in the micro and nanoscale should be optimized by regulating process parameters properly and adopting efficient techniques [1,2].

In order to further optimize these promising micro and nanoscale techniques, both experimental and theoretical studies by using suitable methods are required, enabling a better understanding of material removal mechanisms in the nanoscale. Although a considerable amount of experimental work can be conducted in order to complement theoretical and numerical studies in the microscale, it is difficult or even impossible to conduct experiments and detailed measurements in the nanoscale. A suitable simulation method which can be adopted is the molecular dynamics method (MD). MD was proposed by Alder and Wainwright [3,4] during the 1950s as a computational method capable of simulating material structures and is nowadays established as one of the most appropriate methods for studies in the nanoscale concerning fundamental phenomena relevant to material science and materials processing. Given that the dimensions of MD models are in the range of a few hundred nanometers at most, it is not possible to directly compare the findings of such models with experiments; however, simulations results are still valuable in order to gain sufficient knowledge about fundamental mechanisms of deformation, material removal, and other related phenomena in the nanoscale.

Nano-cutting and nano-abrasive processes have been studied for over three decades using the MD method, beginning with early works such as the ones of Belak and Stowers [5] and Shimada et al. [6]. Although the majority of MD studies are relevant to general nano-machining processes, nano-grinding models have also attracted the attention of various researchers. Initially, the models were smaller and simpler, usually involving a single abrasive grain acting on a monocrystalline metallic or ceramic material; however, nowadays, more sophisticated models have been developed. One of the first MD studies on nanogrinding was conducted by Rentsch and Inasaki [7], who observed the pile-up phenomenon during nanogrinding of copper and made important suggestions for the improvement of MD models, such as employing EAM potential function and proposed methods for reducing calculation speed. Other authors have carried out works regarding the effect of various process parameters during nanogrinding such as grain rake angle, grinding speed, and depth of cut. Komanduri et al. [8] conducted a comprehensive study on the effect of rake angle on cutting force and cutting energy for a broad range of angles, from +45° to −75°. Their findings indicated that although both normal and tangential grinding forces were increased with a decreasing rake angle, the normal force was more considerably increased than tangential forces, whereas friction coefficient increased gradually, reaching a maximum value of 2.361 for a −75°rake angle. Li et al. [9] developed an MD model for high-speed grinding and determined the effect of several important process parameters including grinding speed, depth of cut, and abrasive grain radius regarding chip formation and subsurface damage. Ren et al. [10] investigated the effect of grinding speed and depth of cut on grinding forces, potential, and grinding temperature, and found out that both grinding speed and depth of cut have a positive correlation with all three quantities. Moreover, Liu et al. [11] noted that the effect of grinding speed and depth have a more profound effect of grinding force and temperature rather than stress and specific energy. 

Ren et al. [12] conducted simulations regarding nanogrinding of monocrystalline nickel showing that at low grinding speeds, stacking faults are formed whose size is gradually reduced as speed increases and they are eventually eliminated at very high grinding speeds such as 400 m/s. Furthermore, the increase in both depth of cut and grain size lead to an increase in stacking faults and dislocation in the workpiece while the size of the subsurface damage layer is independent of the values of grinding speed. Zhang et al. [13] also studied the formation of a subsurface damage layer occurring during nano-grinding from the perspective of the final mechanical properties of the workpiece. In their works, they initially investigated the optimum value of grinding speed for effective reduction in the damage layer and afterward determined the mechanical properties of the workpiece by tensile test simulations. The results indicated a relatively weak effect on Young’s modulus but a high impact on workpiece strength after nanogrinding. While in most of the studies, a single grain in a straight path is used for the nano-grinding model, a few researchers developed more advanced models, with complex grain trajectories or a great number of grains. Fang et al. [14] performed nano-grinding simulations with a grain moving at both straight and arc-shaped paths. Zhou et al. [15] created a nano-grinding model in which the abrasive grain moved downwards in an inclined path, then followed a straight path, and finally exited the workpiece in an inclined linear path. Eder et al. [16] conducted nano-polishing simulations with models including multiple abrasive grains with random orientations on a rough surface and even developed a multiscale framework [17] in order to expand their observations regarding the material removal mechanism in larger scales as well. Wu et al. [18] presented a nano-grinding model with two abrasive grains and investigated the effect of the distance between them. However, regarding peripheral nano-grinding [19], research with realistic models is still limited. 

In order to improve machinability, in the case of hard-to-cut materials or to enhance ductile behavior in brittle materials, such as monocrystalline silicon, various hybrid processes are proposed. Heat-assisted machining constitutes a promising technique that was shown to achieve better machinability, favorable surface quality, and lower subsurface damage. This technique has long been applied successfully to material forming processes such as hot forming, performed in elevated ambient temperatures. In the field of nanomachining, a few studies have been conducted, mostly on silicon and related semiconductor materials, e.g., GaAs using the MD method, showing promising results [20,21,22,23]. Most of the studies were conducted by researcher S.Z. Chavoshi and other members of the same research group. Chavoshi and Luo [24] noted an increase in specific cutting energy anisotropy of silicon up to 1400 K and then a decrease for higher temperatures, whereas attrition wear was found to decrease at higher temperatures, contrary to chemical wear. In another study [25], they focused on chip characterization and formation during hot nanomachining, showing that chip ratio and shear plane angle both decreased at higher temperatures, improving machinability. Moreover, it was also found that stresses are considerably reduced at higher temperatures, as well as elastic deformation and that plasticity mode is directionally dependent [26,27]. Regarding plastic flow, it was deduced that at 1200 K, rotational plastic flow is observed in the workpiece and higher vorticity of plastic flow was related to better machinability [28]. Liu et al. [29] underlined that nanomachining at higher temperatures can suppress subsurface damage evolution, change the cutting mode, and enhance the lubricating effect of amorphous layers. Liu et al. [30] found out that nanomachining of silicon at higher temperatures increased plastic deformation and led to the brittle-to-ductile transition. Niu et al. [31] conducted simulations for a wide range of temperatures, from 1 to 1000 K and concluded that the increase in ambient temperature leads to lower forces, higher material removal rate, and lower friction coefficient. Liu et al. [32] studied the hybrid process of thermal and vibration-assisted nanomachining which was proven to result in a reduction in cracks, domination of shearing as cutting mode, and increased number of vacancies in the workpiece. Xie et al. [33] investigated hot nanomachining of γ-TiAl, concluding that this technique led to improved plastic deformation, lower forces and coefficient of friction, increased number of atoms in the high strain zones, and lower dislocation density. Kong et al. [34] performed hot nanomachining simulations of Be in the temperature range of 25–400 °C observing a clear decrease in cutting forces and coefficient of friction values.

As can be seen, the effect of machining at high temperatures for metallic workpieces has not been yet investigated in detail, whereas a considerable amount of work has been conducted on brittle materials. Moreover, these studies usually do not concentrate on nanogrinding, which is a more complex process than nanocutting. Thus, in the present study, an MD model of peripheral nanogrinding using multiple grains with a realistic trajectory was employed to study the effect of nanogrinding at elevated temperatures on grinding forces, chip formation, and subsurface alterations for three different fcc metals, namely, copper, nickel, and aluminum. The results indicated that there is a clear effect on machinability and workpiece deformation during hot nanogrinding for all three materials, depending on their thermal properties, and conclusions were drawn on the suitability of this technique in comparison with other heat-assisted techniques.

## 2. Materials and Methods

**Figure 1:**
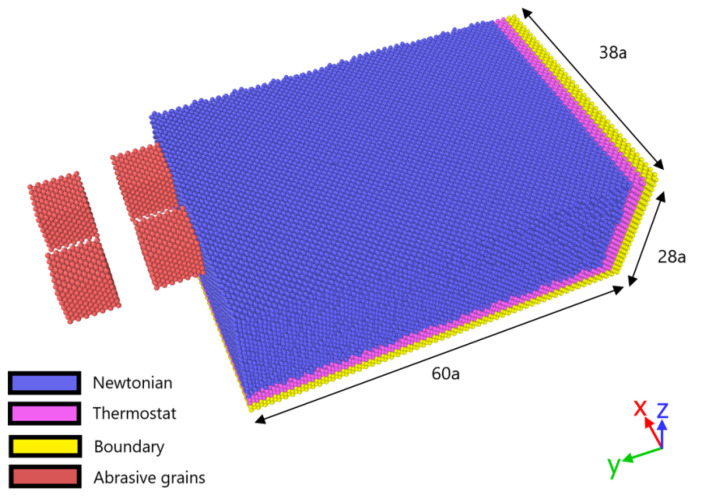
Schematic of the MD model used in the simulations.

In the present work, an MD model of peripheral nano-grinding including multiple abrasive grains is employed in order to conduct simulations regarding hot nanogrinding of various fcc metals, namely, copper (Cu), nickel (Ni), and aluminum (Al). For each metal, a series of hot nano-grinding simulations under various preheating temperatures is conducted. As it is intended to determine the efficiency of this technique and the suitable range of preheating temperatures for each material, simulations under different preheating temperatures are conducted, given that melting temperature, denoted hereafter as T_melt_, is different for each material, especially aluminum, as can be seen in Table 1. Thus, for each material, temperatures up to almost 0.6 T_melt_ are selected. More specifically, for aluminum three different preheating temperature values are selected (400 K, 500 K, and 600 K), as well as for copper (400 K, 600 K, and 800 K), whereas for nickel four different temperatures were selected (400 K, 600 K, 800 K, and 1000 K). The reason for using an additional preheating temperature level for nickel is that its melting point is higher than that of the other two, especially copper, so a temperature higher than 800 K was required to reach approximately 0.6 T_m._ However, it was also intended to determine the effect of a preheating temperature that is lower (400 K) than is common for all three materials. It is to be noted that this range of preheating temperatures is wide enough to allow the thorough study of the correlation between preheating temperature and process outcome such as grinding forces, chip formation, and subsurface damage. All other simulation parameters such as grinding velocity and grinding depth are constant, namely, 100 m/s and 2a_diam_ with a_diam_ denoting the lattice constant of diamond (3.57 Å), and an additional simulation under usual ambient temperature was also conducted in order to assess the differences between nanogrinding at room temperature and under elevated temperatures. The MD nano-grinding model, including various types of atom zones, is presented in Figure 1.

The total grinding length in each case is 160 Å. The grains in the same position in each row have the same protrusion height, with the second row being positioned 1a_diam_ lower in the *z* axis and the spacing between them in the *x* axis is 6a_diam_. Moreover, the two rows of abrasive grains are placed symmetrically with regard to the axis of symmetry of the workpiece in the *x* axis, whereas the two front abrasive grains are positioned at a distance of 1a_diam_ from the workpiece in the *y* axis. The size of the abrasive grains is 6a_diam_. 

In all cases, the workpiece is composed of a single crystal material, with the value of lattice constant in each case presented in Table 1. As all three materials are metals, in order to be able to represent the metallic bond appropriately, taking into consideration the contribution of electron density, for the workpiece material suitable EAM potential functions are selected, which can ensure that the material properties are predicted satisfactorily. EAM potential function includes terms relevant to the electron density, an embedding function, as well as a pairwise potential, as follows:

(1) $$V_{i}(r_{ij})=F_{a}(\sum_{i\neq j}\rho_{\beta }(r_{ij}))+\frac{1}{2}\sum_{i\neq j}\varphi_{\alpha \beta }(r_{ij})$$

In Equation (1), *V_i_* represents the potential energy, *F_a_* is the embedding function, *ρ_β_* represents the electron density, and *φ_α__,β_* represents the pairwise potential whereas *r_ij_* is the distance between atoms *i* and *j*. For the interactions between workpiece material and abrasive grains, namely, Cu–C, Ni–C, and Al–C, Morse potential functions are selected. As diamond is much harder than copper, nickel, or aluminum, the abrasive grains are considered rigid and C–C interactions are neglected. The total number of atoms in the workpiece is 167,000–217,000 depending on the material and the dimensions are 60 × 28 × 38a (in *y*, *z*, and *x* axes, respectively), where a denotes the lattice constant of each material, mentioned in Table 1. The size of the workpiece was deemed sufficient based on preliminary simulations, which showed that the use of periodic boundary conditions and the model length were suitable for reducing any boundary effects and to ensure that the conclusions drawn from the analysis of the simulation results will not be affected by the workpiece size.

The workpiece is composed of three distinct zones of atoms: the boundary zone, the thermostat zone, and the Newtonian zone, depicted in Figure 1 with different colors. The boundary zone of atoms is positioned at the edge of the workpiece and is used in order to prevent rigid body motion of the workpiece and also to reduce boundary effects. The thermostat zone of atoms is positioned above the boundary zone of atoms and is essential in order to dissipate excessive heat from the workpiece by employing a rescaling procedure on these atoms when the temperature exceeded the predefined value. Moreover, the Newtonian zone of atoms constitutes the deformable part of the workpiece in which atoms are free to move under interatomic forces in the workpiece or due to their contact with the abrasive grains. On the YZ plane, periodic boundary conditions are selected so as to simulate the response of a wider workpiece. Furthermore, initial conditions are imposed on atoms according to the Maxwell–Boltzmann distribution for each initial temperature value for a reasonable amount of time for thermalization to occur, and then the nano-grinding process starts with the motion of the abrasive grain. Abrasive grains move at a complex trajectory under the influence of both linear and rotational motion [19], representing the feed of the workpiece and the abrasive wheel rotation with a linear speed of 100 m/s and a surface speed of 108 m/s. The numerical time-step was 1 fs for every simulation and all simulations were carried out using LAMMPS software.

## 3. Results and Discussion

**Figure 2:**
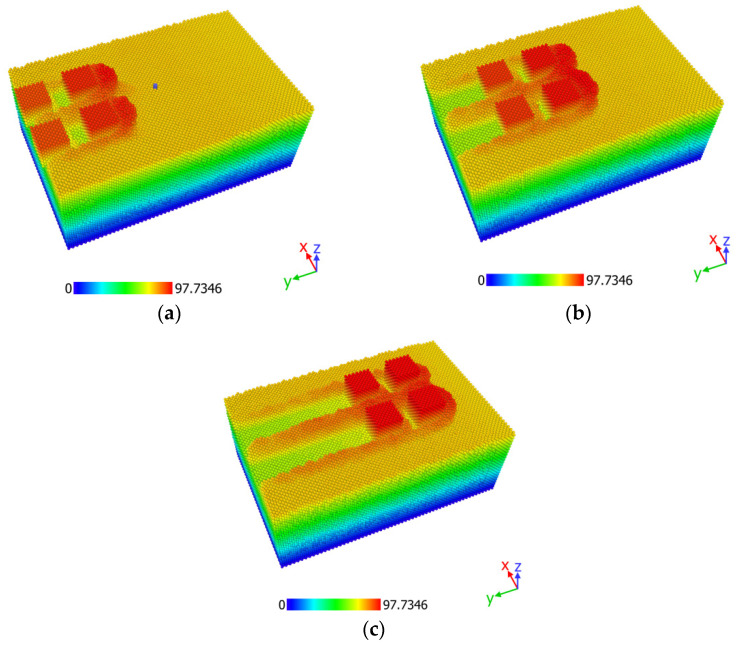
Three snapshots from an indicative nano-grinding simulation (copper, initial temperature: 293 K) at (**a**) 30 ps, (**b**) 50 ps, and (**c**) 80 ps.

After the simulations were conducted, analysis of the obtained results is performed for each different workpiece material. Some indicative snapshots of the simulations are depicted in Figure 2, where the position of each atom in the *z* axis in Å is highlighted. 

Finally, the results are compared to determine whether more general conclusions can be drawn regarding the effect of preheating temperature on fcc metal substrates during hot nanogrinding. 

### 3.1. Hot Nano-Grinding of Copper Workpiece

**Figure 3:**
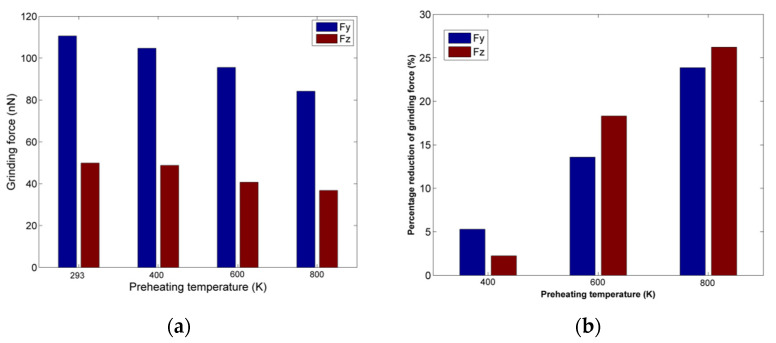
(**a**) Grinding forces values for different preheating temperatures; (**b**) percentage of grinding forces reduction at different preheating temperatures.

The grinding forces results regarding nanogrinding of copper at higher preheating temperature are depicted in Figure 3a. The analysis of grinding forces is essential as forces are an important indicator of machinability. From these results, it can be seen that the preheating temperature plays an important role regarding grinding forces, as force values gradually decrease with an increase in preheating temperature, especially after 400 K, when the decrease is higher. At first, Fy values are more rapidly decreasing in comparison with Fz values but eventually Fz is almost equally affected by the higher preheating temperatures. In order to directly observe the reduction in forces with respect to the case conducted under ambient temperature (293 K), the percentage reduction in each constituent of grinding force is presented in Figure 3b. 

The results depicted in Figure 3b confirm the previous observations, indicating that for Fy a gradual decrease in forces is occurring, whereas for Fz, the increase is larger above 400 K. In general, the decrease in grinding forces at higher preheating temperatures is anticipated as higher workpiece temperature leads to the enhancement of the thermal softening process for the workpiece material, thus lowering its mechanical strength. This effect becomes more pronounced as the preheating temperature exceeds 400 K and finally reaches the higher percentage reduction at 800 K, which is almost 60% of the melting temperature of copper. 

**Figure 4:**
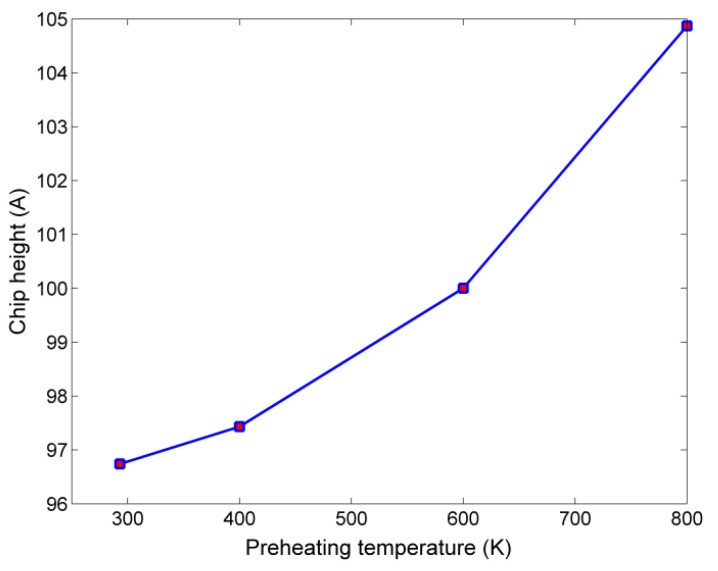
Chip height variation with respect to preheating temperature.

The results regarding chip height variation, during nanogrinding at elevated temperatures are depicted in Figure 4, measured with zero reference point in the bottom layer of the workpiece in the *z* axis. The results of Figure 4 indicatethat a slight increase in chip height occurs, as plastic flow in the workpiece was facilitated by higher preheating temperatures. However, its shape is not considerably altered in respect to the temperature increase, implying that no difference occurred in the cutting mode. 

**Figure 5:**
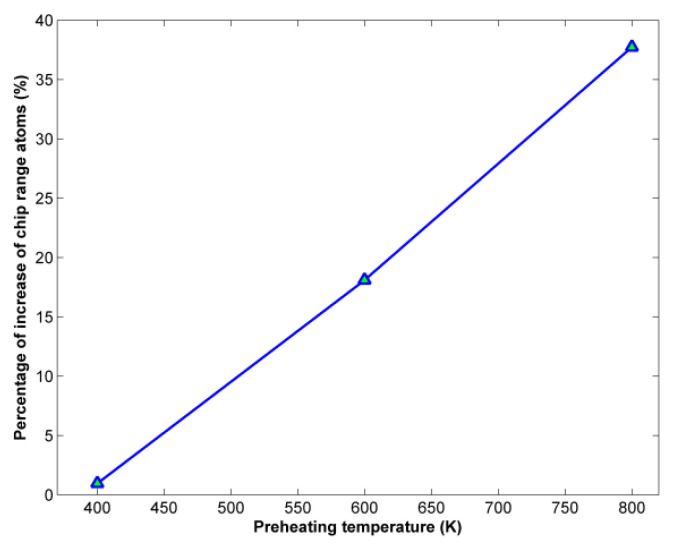
Variation of chip range atoms percentage of increase with respect to preheating temperature.

Moreover, the number of atoms belonging to the chip range was calculated using the methodology described in the work of Uezaki et al. [36], which is relevant to the calculation of displacement vector for the atoms of the workpiece. The results of this analysis, depicted in Figure 5, show that, as the preheating temperature increases, the number of atoms belonging either to the chip or the burr increases too. At lower preheating temperatures, the increase is barely evident but, above 400 K, the number of chip range atoms compared with the number of chip atoms at 293 K increases by more than 20% and eventually by almost 40%. This trend can be explained because due to the decrease in material strength it is possible for more atoms to travel longer distances—in the pile of atoms forming the chip or even being added to the burr—which is formed above the groove walls. Thus, it is determined that higher preheating temperatures can lead to an increase in material removal, something that was also observed in previous works [31]. 

**Figure 6:**
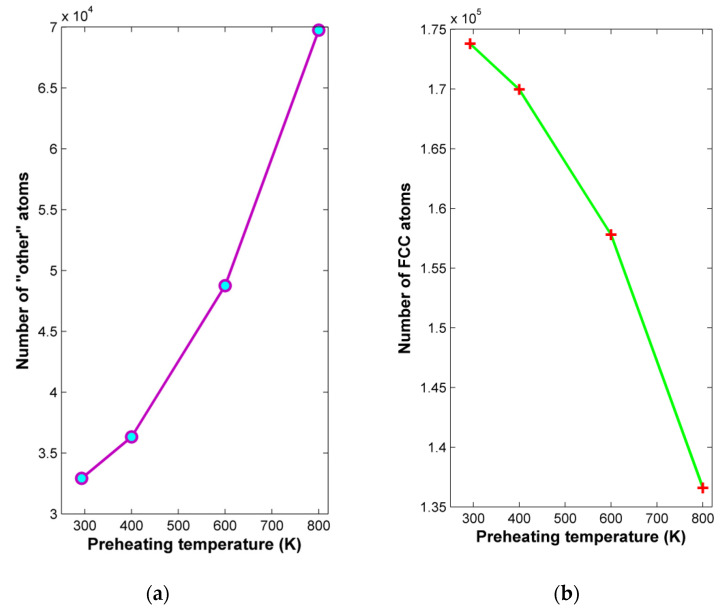
Variation of: (**a**) “other” atoms and (**b**) fcc atoms with respect to preheating temperature.

Apart from the investigations on the effect of preheating temperature on grinding forces and chip characteristics, it is important to observe the effect of preheating temperatures on subsurface structural alterations of the workpiece material for different preheating temperatures. For that reason, the adaptive common neighbor analysis (CNA) method was applied in order to observe the variation of the workpiece structure under different conditions. In Figure 6, the number of atoms belonging to different structures from the fundamental ones, e.g.,fcc, bcc, and hcp, termed as “other” atoms, as well as the number of fcc atoms is depicted with respect to the preheating temperature. From these results, it can be deduced that this parameter has an important impact on workpiece integrity, as the number of “other” atoms increases clearly with increasing preheating temperature values, especially above 400 K. This result can be justified, as higher workpiece temperature leads to lower material strength, enhancing plastic deformation and ultimately leading to easier plastic flow [20,22]. However, for workpiece regions far from the produced grooves, where no material removal takes place and no additional heat due to contact with the abrasive grains is produced, this process resembles thermal processing with a heating and a slow cooling cycle. This condition leads to undesired distortions in the case of high preheating temperatures, something that contributes to the higher number of “other” atoms in these cases.

### 3.2. Hot Nano-Grinding of Nickel Workpiece

**Figure 7:**
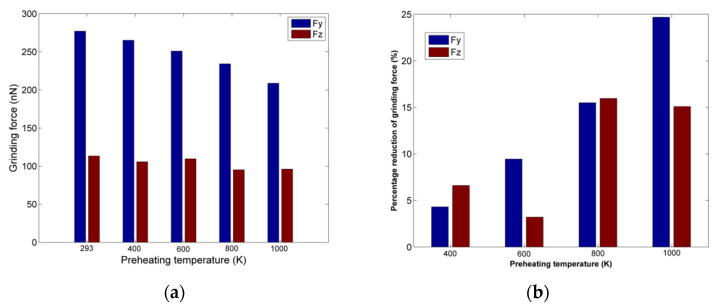
(**a**) Grinding forces values for different preheating temperatures; (**b**) percentage of grinding forces reduction at different preheating temperatures.

The grinding forces results regarding nanogrinding of nickel at higher preheating temperature are depicted in Figure 7a. As the melting temperature for nickel is higher than copper, apart from preheating temperatures of 400 K, 600 K, and 800 K, a simulation under preheating temperature of 1000 K was also carried out. As in the case of copper nanogrinding, the decrease in grinding forces with increasing preheating temperatures is clear, but this decrease is not so important before 600Κ. Moreover, Fy is gradually decreasing monotonically, whereas values of Fz are practically stabilized above 800 K.

An interesting result is observed in Figure 7b, regarding the percentage of force decrease in each case. Contrary to the results regarding copper, the decrease is not so important for preheating temperatures lower than 600 K; however, the percentage decrease exceeds 15% for both forces for temperatures higher than 800 K, leading eventually to a decrease of 25% for Fy. These observations suggest a probable correlation of the force reduction trend with the thermal properties of each material, especially melting temperature, something which will be further analyzed in Section 3.4.

**Figure 8:**
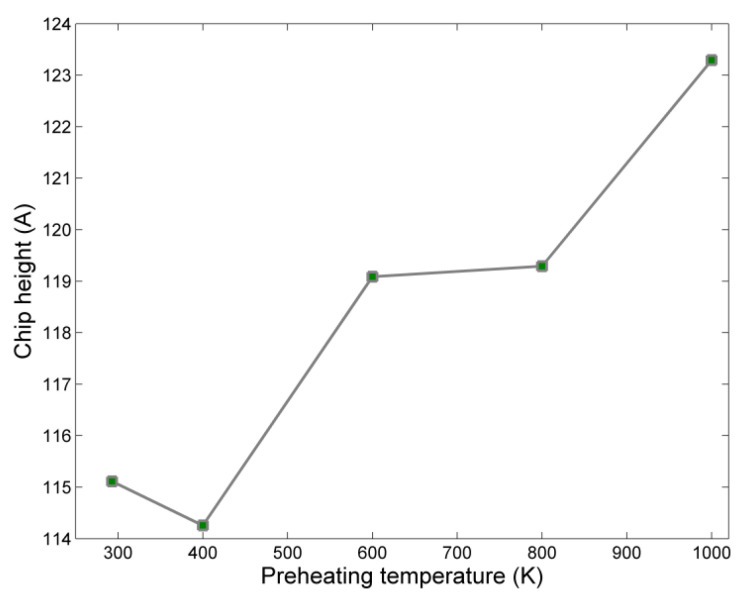
Chip height variation with respect to preheating temperature.

Regarding variation of chip height, it can be seen from Figure 8 that a small increase in chip height occurs with increasing preheating temperature. Initially, a small decrease is observed, and then the chip height increases gradually with a greater increase between the cases with preheating temperature values of 400 and 600 K, as well as between 800 and 1000 K. The larger values of chip height obtained only at higher temperatures can probably be attributed to the fact that up to 600 K no significant impact of preheating temperature is observed to the workpiece, which was also observed regarding grinding force components.

**Figure 9:**
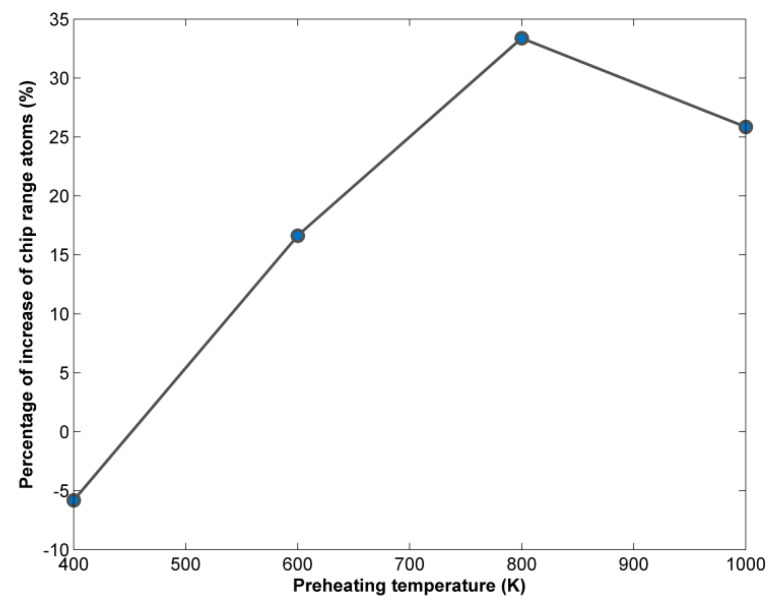
Variation of chip range atoms percentage of increase with respect to preheating temperature.

Furthermore, the percentage of increase in the number of chip range atoms in each case compared with the number of chip range atoms at 293 K was calculated in a similar way as in the previous subsection. The results of this analysis show that the higher preheating temperature leads, initially, to a small decrease in the number of atoms belonging either to the chip or the burr, and afterward the number of chip range atoms increases until 800 K, with a small decrease being observed at 1000 K. Some differences in the trends of chip height and number of chip range atoms in the case of the nickel workpiece material are attributed to a particular characteristic of material removal in these cases. In particular, due to the higher forces between grains and workpiece, more material is removed in the region between the grains than in the cases of copper and aluminum workpieces, leading to a larger chip and thus, the graphs in Figure 8 and Figure 9 do not exactly correspond to those of Figure 4 and Figure 5 (or Figures 12 and 13). However, the general trend regarding the increase in chip height and volume is common in all cases.

**Figure 10:**
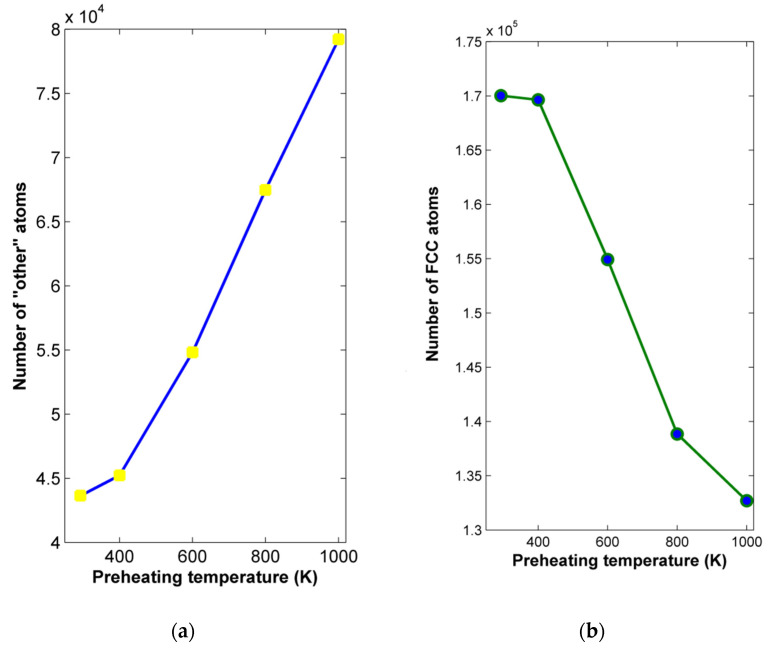
Variation of: (**a**) “other” atoms and (**b**) fcc atoms with respect to preheating temperature.

Finally, it is also necessary to investigate the effect of preheating temperature on subsurface structural alterations of the nickel workpiece for different preheating temperatures. Using an adaptive CNA method, the number of “other” atoms as well as the number of fcc atoms were determined with respect to preheating temperature and are depicted in Figure 10. From the results of Figure 10a, it can be seen clearly that although the increase in preheating temperature leads clearly to an increase in the number of “other” atoms in the workpiece, the increase is steeper only for temperatures over 400 K and even steeper above 800 K. This result can be justified due to the superior strength of nickel compared with copper at elevated temperatures. However, it was again shown that the increase in preheating temperature has an adverse effect on workpiece integrity, something that should be properly managed.

### 3.3. Hot Nano-Grinding of Aluminum Workpiece

**Figure 11:**
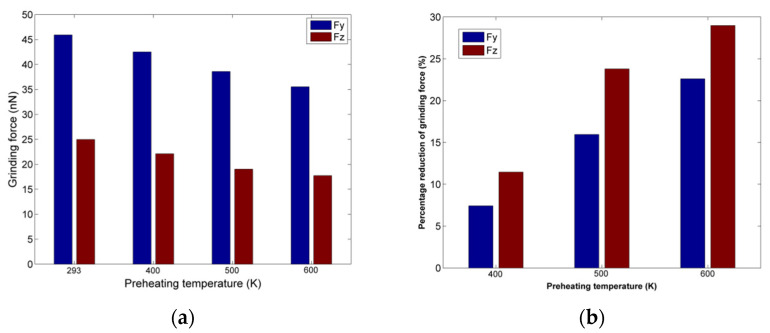
(**a**) Grinding forces values for different preheating temperatures; (**b**) percentage of grinding forces reduction at different preheating temperatures.

The grinding forces results regarding nano-grinding of aluminum at different preheating temperatures are depicted in Figure 11a. As the melting temperature for aluminum is much lower than for copper, simulations at preheating temperatures of 400 K, 500 K, and 600 K, were carried out. Furthermore, as in the case of the other two materials, the decrease in grinding forces with increasing preheating temperatures is again clear, with considerable decrease being observed even at 400 K, especially for Fy. Moreover, both Fy and Fz decrease monotonically with increased preheating temperature.

The results regarding the percentage of force decrease, depicted in Figure 11b, reveal that both forces decrease in each case, confirming the observations from Figure 11a. Contrary to the results regarding copper or nickel, the decrease is important for lower preheating temperatures, as the percentage decrease exceeds 20% for Fz at 500 K and both grinding forces are reduced by over 20% for temperatures higher than 500 K. Thus, this different behavior can be associated with the thermal properties of aluminum and especially the relatively low melting temperature of aluminum, which leads to a significant decrease in strength.

**Figure 12:**
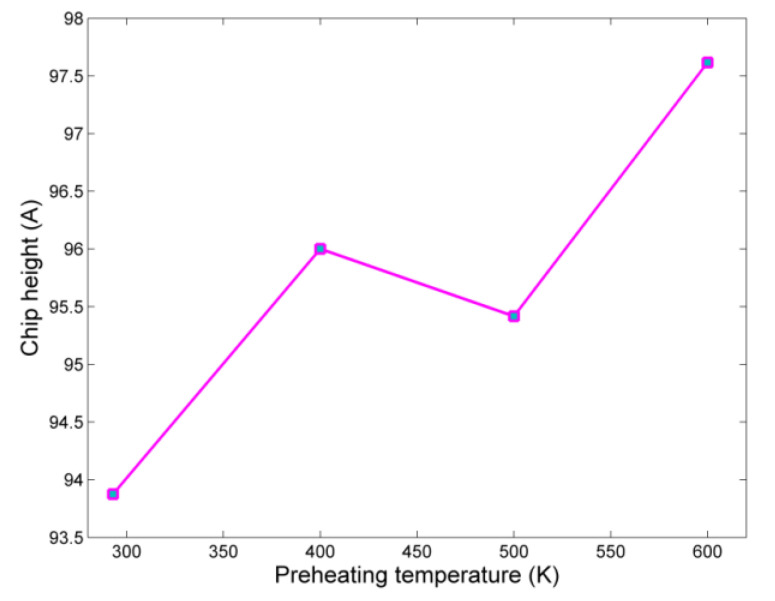
Chip height variation with respect to preheating temperature.

**Figure 13:**
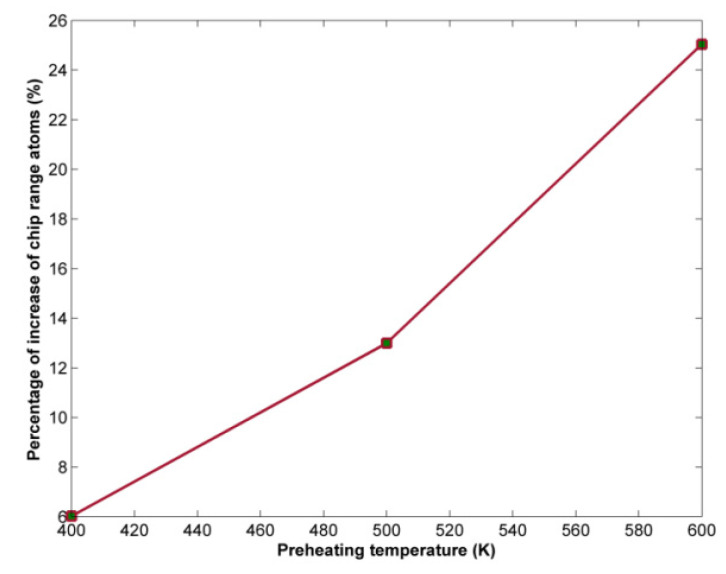
Variation of chip range atoms percentage of increase with respect to preheating temperature.

The variation of chip height in the cases of aluminum nanogrinding is depicted in Figure 12. These results also indicate that the impact of preheating temperature is relatively low, although in general, at higher preheating temperatures a slight increase in chip height occurs. Afterwards, the number of chip range atoms in each case was calculated. The results of the analysis of the percentage increase in the number of chip range atoms indicate, again, that the higher preheating temperature leads gradually to an increase in the number of atoms of the chip or the burr. The major increase in the number of chip range atoms occurs between 500 K and 600 K (Figure 13).

**Figure 14:**
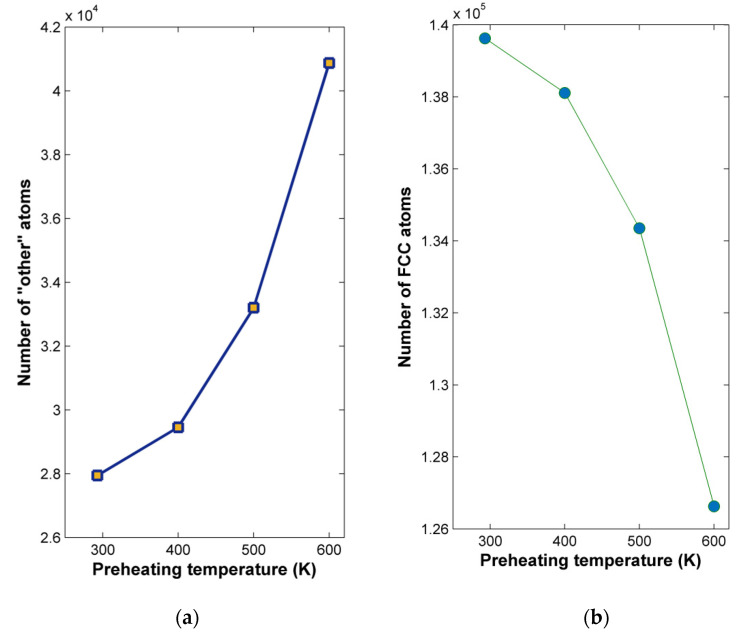
Variation of: (**a**) “other” atoms and (**b**) fcc atoms with respect to preheating temperature.

Finally, the effect of preheating temperature on subsurface structural alterations of the aluminum workpiece with respect to preheating temperature is investigated. In Figure 14, the results of CNA indicate that the number of “other” atoms increases gradually as the preheating temperature increases and this trend becomes steeper at higher preheating temperatures. As in the case of the two other workpiece materials, it was observed that the increase in preheating temperature has an important effect on workpiece integrity.

### 3.4. Comparison of the Effect of Preheating Temperature on Different Materials

**Figure 15:**
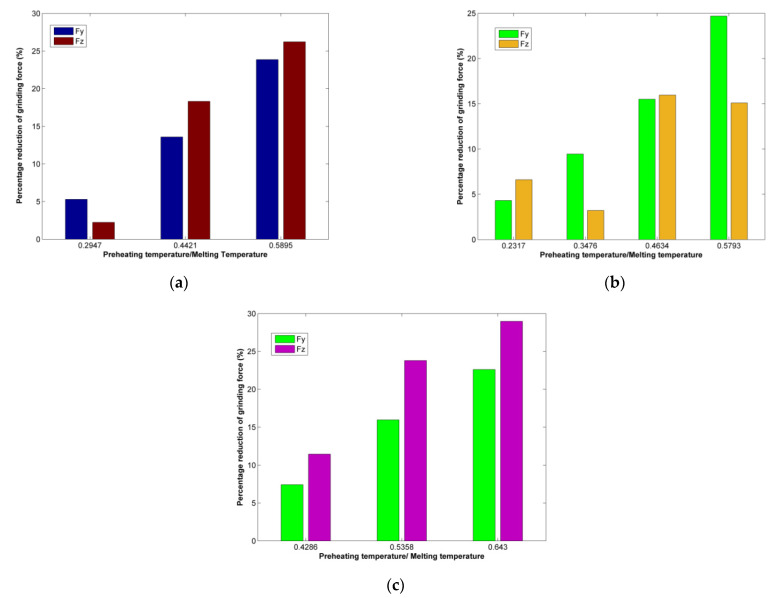
Percentage of grinding forces reduction at different preheating temperatures to melting temperature ratio values: (**a**) copper workpiece, (**b**) nickel workpiece, and (**c**) aluminum workpiece.

After the determination of the effect of preheating temperature during nanogrinding was performed on each different material, it is worth discussing some important findings which are common in all cases. At first, regarding grinding force, it can be seen from Figure 15 that although the preheating temperature affects to a different extent each different workpiece material or different components of grinding force, it is observed that the percentage of grinding forces decrease is similar if the temperatures are compared with the melting temperature of each material, especially for the Fy component. For example, at around 0.58 T_melt_, Fy is reduced by almost 25% for both copper and nickel workpieces, whereas the decrease is slightly lower for the aluminum workpiece. On the other hand, for temperatures below 0.3 T_melt_, the decrease in both copper and nickel workpieces is only around 5%. From these observations, it can be concluded that a value of preheating temperature at least around 0.4–0.45 T_melt_ is suggested for a meaningful effect on grinding forces. 

Although it is not possible to directly compare the results of the simulations with experiments of the same scale, the observed trends can be compared qualitatively with those of similar works in the relevant literature. By analyzing the results of previous works on the effect of preheating temperature during nanomachining, it was found that the observations of the current work are consistent with findings in the relevant literature [20,21,22,23,25,30,31,33,34]. More specifically, in works regarding hot nanomachining, similar percentage decreases were observed. Although, for semiconductor materials, the decrease was usually lower in comparison with the preheating temperature to melting point ratio [24,25,30], and in a few cases, such as during the nanocutting of Be [34], a larger decrease in cutting forces (32–36%) was observed at a moderate ratio (0.43 T_melt_).

As it was observed that the use of preheating has an adverse effect on workpiece integrity, which is a crucial parameter, the results from nano-grinding simulations of different materials will be compared to determine possible common characteristics. The most abrupt increase in the number of “other” atoms was observed over 600 K for the copper workpiece, over 800 K for the nickel workpiece, and over 500 K for the aluminum workpiece. Thus, it is suggested that the preheating temperature should not exceed 0.5–0.55 T_melt_ at most, in order to avoid high levels of deformation in the workpiece. Taking into consideration the reduction in grinding forces as well, the preheating temperature for fcc metals should be selected in the range of 0.4–0.55 T_melt_, depending on other thermal properties of each material, such as thermal conductivity and specific heat, as well.

Moreover, it can be observed that similar levels regarding the increase in the number of chip range atoms at high preheating temperatures occur for copper and nickel, namely, between 30–35%, whereas for aluminum the increase is smaller, with a maximum value around 25%. In these cases, an increase in material removal rate is noted, which was also reported in the relevant literature [22,31].

## 4. Conclusions

In the present work, for the first time, the effect of preheating temperature on nanogrinding of various monocrystalline fcc metals, namely, copper, nickel, and aluminum was investigated in order to determine the efficiency of applying this technique regarding the reduction in grinding forces while maintaining acceptable levels of deformation and structural integrity in the workpiece;thus expanding the knowledge in the relevant scientific field. For that reason, an MD peripheral nano-grinding model is developed with multiple grains at a non-linear, realistic trajectory taking into consideration that various possible mechanisms such as rubbing, ploughing, and cutting can occur during nanogrinding due to the variable chip thickness, which is usually not considered in the relevant literature. Simulations using this model are carried out under different preheating temperatures for all three materials. From the findings of the study, several conclusions can be drawn.

Regarding grinding forces, the decrease in force components is evident as preheating temperature is higher, due to the reduction in material strength and facilitation of plastic flow. However, it was observed that the effect of preheating temperature can be correlated with thermal properties of the substrate materials, especially melting temperature, as for the same process parameters, a similar percentage reduction (up to 25–30%) of grinding forces was noted at around 60% of the melting temperature of the workpiece material. Moreover, based on these findings, it is suggested that in order to observe an effective reduction inforces, preheating temperature at least around 0.4–0.45 Τ_melt_ should be selected. 

Regarding chip formation, the increase in preheating temperature led to a slight increase in chip height, between 3.5 to 9 Å depending on the workpiece material, probably due to higher deformation. Moreover, a slight increase in the number of chip range atoms was also observed, between 25–35% depending on the workpiece material, indicating a relatively higher material removal rate in cases with higher preheating temperatures due to lower material resistance.

Regarding subsurface damage, CNA method results indicated that the number of “other” atoms belonging to a different structure than the initial substrate structure increases considerably with increasing temperature; an increase of up to two-fold for copper workpieces, especially for temperatures above 0.5–0.55 T_melt_, due to a higher degree of material strength reduction, allowing for easier deformation. Thus, in order to achieve both low grinding forces and acceptable levels of workpiece integrity, the preheating temperature for fcc metals should be in the range of 0.4–0.55 T_melt_. Finally, although the application of the preheating technique was effective for reducing grinding forces and facilitated material removal during nanogrinding, it can also considerably increase the deformation of the substrate, which could be avoided if a high power thermal beam, e.g., a laser was used instead, affecting mainly the region where material removal takes place. 

## Figures and Tables

**Table 1:** Properties of materials used in the simulations [35].

Material	Lattice Constant (Å)	Density (kg/m^3^)	Thermal Conductivity (W/mK)	Specific Heat (J/kgK)	Melting Temperature (K)
Copper	3.615	8.954	386	380	1357.16
Nickel	3.520	8.906	99	445.9	1726.16
Aluminum	4.041	2.707	220	896	933.16

## Data Availability

Not applicable.
